# Socio-economic and cultural factors associated with the utilization of maternal healthcare services in Togo: a cross-sectional study

**DOI:** 10.1186/s12978-023-01644-6

**Published:** 2023-07-25

**Authors:** Komlan Kota, Marie-Hélène Chomienne, Robert Geneau, Sanni Yaya

**Affiliations:** 1 Faculty of Health Sciences, Interdisciplinary School of Health Sciences, University of Ottawa, Ottawa, ON Canada; 2 Department of Family Medicine, Faculty of Medicine, University of Ottawa, Ottawa, Canada; 3 Applied Research Division, Public Health Agency of Canada, Ottawa, Canada; 4 School of International Development and Global Studies, University of Ottawa, Ottawa, ON K1N 6N5 Canada; 5 The George Institute for Global Health, Imperial College London, London, UK

**Keywords:** Maternal health, Togo, Antenatal care, Facility delivery, Healthcare utilization, Global health

## Abstract

**Background:**

Proper utilization of maternal healthcare services plays a major role on pregnancy and birth outcomes. In sub-Saharan Africa, maternal and child mortality remains a major public health concern, especially in least developed countries such as Togo. In this study, we aimed to analyze factors associated with use of maternal health services among Togolese women aged 15–49 years.

**Methods:**

This study used data from third round of nationally representative Demographic and Health Survey conducted in Togo in 2013. Analysis included 4,631 women aged 15–49 years. Outcome variables were timely first antenatal care (ANC) visits, adequate ANC4 + visits, and health facility delivery. Data were analyzed using Stata version 16.

**Results:**

Overall, proportion of maternal healthcare utilization was 27.53% for timely first ANC visits, 59.99% for adequate ANC visits, and 75.66% for health facility delivery. Our multivariable analysis showed significant differences among women in highest wealth quintile, especially in rural areas with increasing odds of timely first ANC visits (Odds ratio (OR) = 3.46, 95% CI = 2.32,5.16), attending adequate ANC visits (OR = 2.19, 95% CI = 1.48,3.24), and delivering in health facilities (OR = 8.53, 95% CI = 4.06, 17.92) compared to those in the poorest quintile. Also, women with higher education had increased odds of timely first ANC visits (OR = 1.37, 95% CI = 1.11,1.69), and attending adequate ANC visits (OR = 1.73, 95% CI = 1.42,2.12) compared to those with no formal education. However, having higher parity and indigenous beliefs especially in rural areas decreased odds of using healthcare services.

**Conclusions:**

Findings from this study showed that socio-economic inequality and socio-cultural barriers influenced the use of maternal healthcare services in Togo. There is therefore a need to improve accessibility and the utilization of maternal healthcare services through women’s economic empowerment and education to reduce the barriers.

## Plain language summary

Utilization of healthcare services by women of childbearing age has been shown to reduce maternal and child mortality. However, in sub-Saharan Africa, maternal and child mortality remains a major public health issue. This is especially the case in Togo, one of the smallest countries in West Africa. The objective of this study was to assess the factors associated with utilization of maternal healthcare services among Togolese women aged 15–49 years. This was a cross-sectional study that used data from the Togo 2013 Demographic and Health Survey, a nationally representative household survey of women of childbearing age in Togo. Findings from the current study showed that the overall proportion of maternal healthcare utilization was 27.53% for timely first antenatal care visits, 59.99% for adequate antenatal care visits, and 75.66% for health facility delivery. Also, this study found that socio-economic inequality and socio-cultural barriers influenced the use of maternal healthcare services in Togo. For instance, women in the highest wealth quintile, those with higher education, and those who were covered by health insurance had higher odds of utilizing maternal healthcare service. Whereas, women who had indigenous beliefs, those from other ethnicities, who lived in the Savanes and Plateaux regions, as well as those with higher parity had lower odds of using maternal healthcare services. The results indicate that there is an urgent need to promote women’s economic empowerment and education to improve accessibility and the utilization of maternal healthcare services.

## Background

In 2017, according to the World Health Organization (WHO) report, more than 295 000 women died worldwide from preventable causes due to pregnancy or childbirth, which represented approximately an average of 810 maternal deaths per day [1, 2]. Most of these maternal deaths (94%) occurred in low- or middle-income countries (LMICs), especially in sub-Saharan Africa where two-thirds (66%) of global maternal deaths are reported [2, 3]. Despite more than a 38% decline in maternal deaths globally, from 342 to 211 deaths per 100,000 livebirths, between 2000 and 2017, the maternal deaths ratio is still unacceptably high [2, 4]. Also, still between 2000 and 2017, the sub-Saharan African maternal mortality ratio stood at 542 maternal deaths per 100,000 livebirths higher than the global ratio of 216 deaths per 100,000 livebirths [3, 5].

To prevent and reduce these maternal deaths, numerous interventions have been developed. One of such interventions is the UN’s adoption of Sustainable Development Goals (SDGs)—the target of one of the SDG-3 is reducing the global maternal deaths to less than 70 deaths per 100,000 livebirths by 2030 [6, 7]. Also, in 2021, five new targets were established to help countries get back on track in decreasing maternal deaths and tracking progress against the SDGs. Of these, the first three targets requires that 90% of pregnant women must attend ANC4 + visits, 90% of births are attended by skilled health personnel and 80% of women who gave birth must have accessed prenatal care (PNC) [7]. Despite these, the world is not on pace to meet the SDG-3 target of reducing maternal deaths [7] and low maternal health care service utilization remains a public health concern for LMICs, due to inequalities in access to quality health services, limited skilled health personnel and the wide gap between the rich and the poor which has increased the healthcare systems challenges [2, 8].

In sub-Saharan Africa for example, only 43% of pregnant women received ANC4 + visits compared to the global average of 55%, and 49% of deliveries are attended by skilled health personnel compared to the global average of 70% [9]. In Togo, physical access to care is rated very low for nearly all regions of the country, except for Lomé where the performance of the health system is acceptable. The lack of health infrastructure and limited physical access to health facilities is a major barrier to the use of maternal health services in this country [10, 11]. A third (33%) of people live outside a radius of 5 km from a health facility and there is a lack of ambulances (0.22 ambulances per health facility) with a weak referral systems [12]. In addition to the weak infrastructure (lack of electricity, water, drugs, and equipment), the skills of some health professionals are weak due to the low quality of training, poor working conditions, and unequal distribution of personnel over the national territory [12].

A number of studies have documented that socio-economic inequalities are implicated in the underutilization of maternal healthcare services [13, 14]. For instance, evidence indicates that socio-economic barriers are closely linked to the basic health coverage of the population which remains very low in Africa [15, 16]—only 48% of the continent’s population have access to the healthcare services they need [17, 18]. In Togo, only 7.6% of the population was covered by a health system of financial risk protection in 2016 compared to an average of 17% in sub-Saharan Africa, and 51% of health spending was supported by households through direct payments [19]. Direct payments for health remains very significant and are often a source of impoverishment of the population [12, 19].

Finally, apart from socio-economic inequalities and physical barriers, sociocultural factors may also limit access to maternal health care services. This includes ethnicity, religion, household decision-making, gender and autonomy, information, and education. These sociocultural factors pose significant barriers in Africa [20, 21]. In fact, they vary considerably not only between individual countries but also between different communities. For instance, in Burkina Faso, key predictors of home births are distance from the household to the health center, prenatal visits, prior experience of giving birth at home, fear of caesarean delivery, and lack of transport to go to health center [22]. This shows that there are many factors that determine access to maternal services. Some are common to many of the countries, but specificities exist. A specific study adapted to the context of each country on the determinants of the use of maternal health services is essential to identify them, consider corrective policy actions and suggest the appropriate strategies. This study was therefore undertaken to analyze the determinants affecting the use of maternal health services among Togolese women aged 15 to 49 years.

## Methods

### Study setting

Togo is one of the smallest countries in West Africa, with a land surface area of 56,785 km^2^ and an estimated population of 8,848,699 of which majority are females [23].

According to the World Bank (2022), less than half of the population live in urban areas, over 50% of the population are Christians and about 33% have indigenous beliefs [23]. Over half of the population live on less than $1.90 a day despite the country’s economic growth of 4.4% and global efforts made by the government to attract investment and encourage development [24].

### Data source and sampling technique

In this study, we used secondary data from the most recent 3^rd^ Togo Demographic and Health Survey (TDHS-3) which was conducted between November 2013 to April 2014. This cross-sectional survey was carried out by the National Institute of Statistics Economical Studies and Demographic (INSEED) in collaboration with the Ministry of Health (MoH) of Togo. The survey was financed by the United States Agency for International Development (USAID) and other international donors with the technical assistance of the International Coaching Federation (ICF) International, Inc.

The 2013 TDHS-3 employed a stratified two-stage cluster design to select the study participants. In the first stage, 330 primary survey units or clusters (128 in urban areas vs 202 in rural areas) were systematically selected with probability proportional to the unit size from the list of Enumeration Areas established during the General Census of Population and Housing conducted in 2010 by the INSEED. In the second stage, a sample of 30 households were selected systematically with equal probability from the list of households in each primary unit [25]. In this survey, 9697 eligible women aged 15–49 years were identified for an interview from 9549 households interviewed, of whom 9,480 women were successfully interviewed with a response rate of 98% (urban 97 vs. 99% rural). For this study, only participants who provided data on maternal healthcare utilization were selected for analysis. The unit of analysis for this study were individual women who were of childbearing age in Togo. Further details of the survey have been published elsewhere [25].

## Variables

### Outcome variables

The dependent variables in this study were timing of the first ANC visit, adequate number of ANC visits, and place of delivery.

### Measurement of variables

Timing of the first ANC visit is defined as attending the first ANC visit within the first three months of pregnancy. This variable was coded as “1” (early initiation) if the participants reported attending the first ANC visit within the first trimester and “0” (late initiation) if after three months of conception [26, 27].

Adequate ANC visits are defined as receiving at least four ANC visits during the last pregnancy. Adequacy of ANC was coded as “1” (adequate) if participants had ANC4 + visits during their last pregnancy and “0” (inadequate) if less than four visits [28–30].

Place of delivery is categorized as health facility (public or private hospital, clinic, medico-social center, community health center) and “home” if the childbirth took place at respondent’s home [31, 32].

### Explanatory variables

Our independent variables were the sociodemographic characteristics of the participants which included age (15–24, 25–34, and 35–49), education status (No education, primary, secondary/higher), religion (Christian, Muslim, indigenous believer), ethnicity (Adja-ewe/Mina, Kabye/Tem, Akposso/Akebou, Ana-ife, Para-gourma/Akan, Other and Non-Togolese), type of residence (urban or rural), region (Grande Agglomeration de Lomé, Maritime, Plateaux, Centrale, Kara and Savanes), health insurance (No and Yes), parity (None, 1–2, 3–4 and > 4), last child wanted (No and Yes), currently employed (No and Yes), and wealth index (poorest, poorer, middle, richer and richest).

### Statistical analyses

Data analysis was performed using Stata version 16 statistical software. Because of the clustered nature of the data, we used complex sampling method, taking into consideration sampling strata, weight, and primary sampling units by using the svy (survey) command. In the first step, we used descriptive analysis to calculate the frequencies, percentages, and cumulative percentages for explanatory variables. In the second step, bivariate and multivariable analyses were conducted. The bivariate analysis comprised of sociodemographic characteristics as explanatory variables and use of maternal healthcare services as the outcome variables. Unadjusted odds ratios, 95% confidence intervals and p-values were calculated and all explanatory variables that were significant (with p-values < 0.05) were considered as having a potential association with the outcome variables and were selected and added to the multivariable logistic regression model.

The multivariable analyses were performed to calculate the odds of the association between the use of maternal healthcare services (outcome variables) and socio-demographic characteristics (explanatory variables) that were significant in the bivariate analysis. Adjusted odds ratios, 95% confidence intervals and p-values were also computed and all explanatory variables with a p-value < 0.05 in the multivariable analysis were considered as having a statistically significant association with use of maternal healthcare services (outcome variables). Three outcome variables were presented in tables, each divided into two subsamples: urban and rural. For all regression analyses, the odds ratios (OR) were presented along with their corresponding 95% Confidence Interval (CIs), and p-values. We also checked for multicollinearity using the VIF (Variance inflation factor) method, and the value was < 10 (Mean = 1.74, Minimum = 1.03, Maximum = 3.72) which indicated no multicollinearity among the variables. Associations with p-values of < 0.05 were considered statistically significant.

### Ethical considerations

This study used secondary data which is available in the public domain (https://dhsprogram.com/data/dataset/Togo_Standard-DHS_2013.cfm?flag=0); no further ethical procedures were therefore required for this study.

## Results

### Socio-demographic characteristics of the respondents

Table 1 shows the characteristics of the women who participated in the present study (n = 4631). Two thousand two hundred and twenty-two (47.98%) of the participants were in the age-group of 25–34 years, over two-fifth (41.48%) had no formal education, and about half (50.81%) were Christians. More than a quarter (29.02%) of the participants were from the Para-gourma/Akan ethnicity, most of them (68.34%) lived in rural areas and about one-fifth (22.95%) were from Savanes region. The majority of the women (96.07%) reported having no health insurance, more than two-fifth (40.36%) had a parity less than three and over than nine-tenths (93.44%) reported wanting their last child. More than four fifth (83.24%) had occupations and over a quarter (28.01%) were from household in the poorest wealth quintile.

**Table 1:** Socio-demographic characteristics (n = 4631)

Variable	Freq.	Percent (%)
Age-groups		
15–24	1067	23.04
25–34	2222	47.98
35–49	1342	28.98
Education		
No Education	1921	41.48
Primary	1617	34.92
Secondary and higher	1093	23.60
Religion		
Christian	2353	50.81
Muslim	984	21.25
Indigenous beliefs	1294	27.94
Ethnicity		
Adja-ewe/Mina	1333	28.78
Kabye/Tem	1292	27.90
Akposso/Akebou	178	3.84
Ana-ife	127	2.74
Para-gourma/Akan	1344	29.02
Other	132	2.85
Non-togolese	225	4.86
Place of residence		
Urban	1466	31.66
Rural	3165	68.34
Region		
Grande Agglomération de Lomé	943	20.36
Maritime	476	10.28
Plateaux	819	17.69
Centrale	678	14.64
Kara	652	14.08
Savanes	1063	22.95
Health insurance		
No	4449	96.07
Yes	182	3.93
Parity		
1–2	1869	40.36
3–4	1345	29.04
> 4	1417	30.60
Last child wanted		
No	304	6.56
Yes	4327	93.44
Currently employed		
No	776	16.76
Yes	3855	83.24
Wealth index		
Poorest	1297	28.01
Poorer	867	18.72
Middle	879	18.98
Richer	801	17.30
Richest	787	16.99

**Fig. 1:**
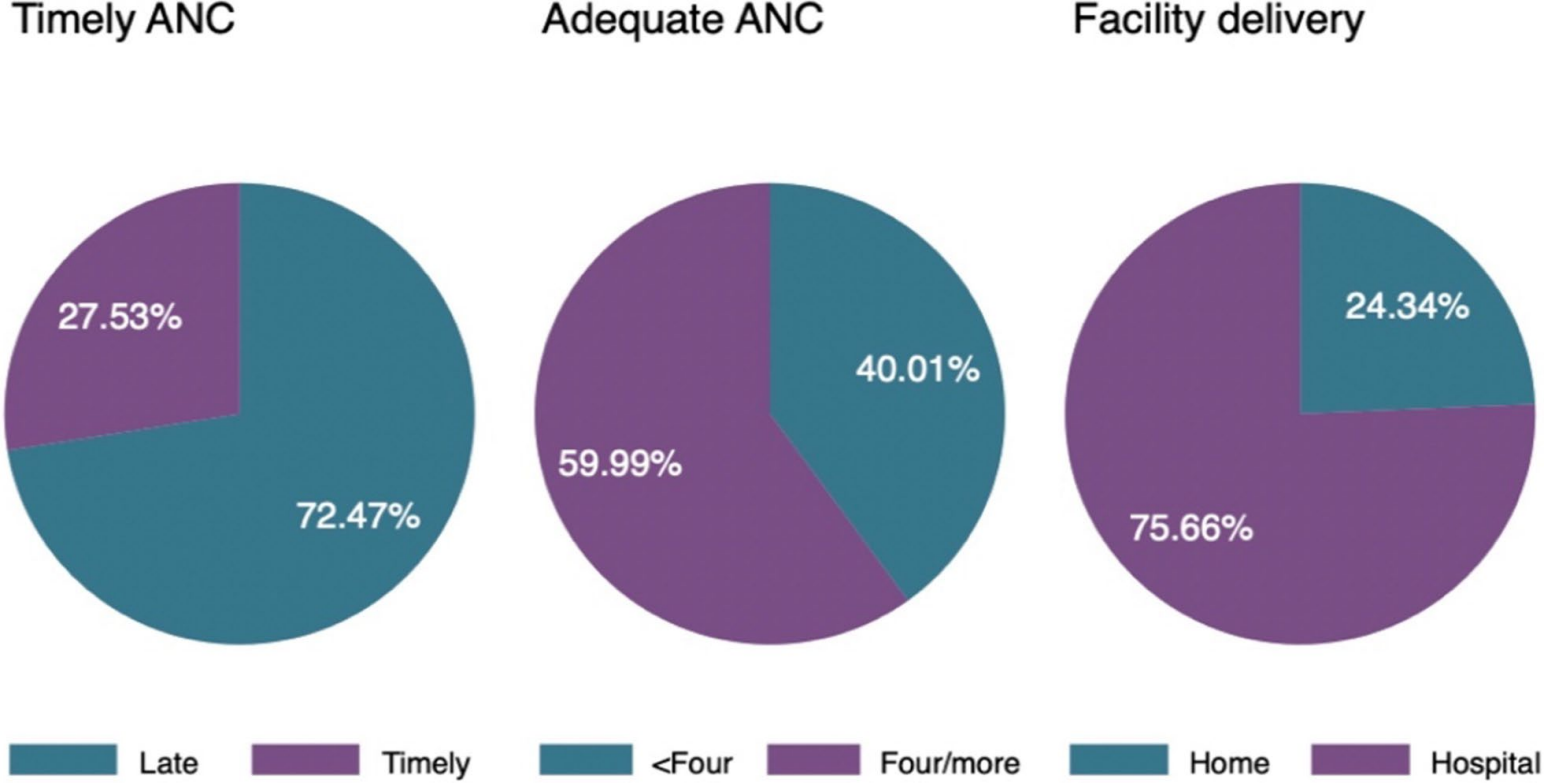
Percentage of timely ANC visits, Adequate ANC visits and facility

Figure 1 shows that nearly three quarters of the women (72.47%) attended their first ANC visit late, about three-fifth (59.99%) had made an adequate number (four or more) of ANC visits during their pregnancy, and about three quarters (75.66%) gave birth in a hospital.

### Factors associated with timing of first ANC visits in Togo

Table 2 shows the factors associated with timing of first ANC visits. In the bivariate analysis, all variables that had p-values < 0.05 were selected and added to the multivariable logistic regression model. The findings showed that women with secondary and higher education had higher odds of timely first ANC visit (OR = 1.37, 95% CI = 1.11,1.69) compared to those with no formal education. Stratified by areas, this positive association was true for urban women only (OR = 1.67, 95% CI = 1.20,2.33). For place of residence, rural women had increased odds of timely first ANC visits (OR = 1.61, 95% CI = 1.16,2.22), compared to those living in urban areas (Table 2). Also, women who were covered by health insurance had increased odds of early first ANC visits (OR = 1.93, 95% CI = 1.39,2.67). Upon stratification by areas, this association was significant for both urban (OR = 2.07, 95% CI = 1.33,3.22) and rural (OR = 1.69, 95% CI = 1.02,2.79) areas. In addition, women who were from the rich and richest wealth quintile, especially in the rural areas had increased odds of timely initiation of ANC visits (OR = 1.90, 95% CI = 1.35,2.67) and (OR = 3.46, 95% CI = 2.32,5.16) compared to those from the poorest wealth quintile.

**Table 2:** Factors associated with timing of first ANC in Togo

	Bivariate analysis (OR, 95%CI)			Multivariable analysis (OR, 95%CI)		
	Overall	Urban	Rural	Overall	Urban	Rural
Timing of first ANC visits						
Age groups (15–24)	Ref.	Ref.	Ref.			
25–34	1.06 [0.90,1.24]	0.98 [0.75,1.27]	1.00 [0.80,1.24]			
35–49	1.02 [0.85,1.22]	0.98 [0.72,1.33]	1.12 [0.89,1.41]			
Education (no education)	Ref.	Ref.	Ref.	Ref.	Ref.	Ref.
Primary	1.50^***^ [1.29,1.76]	1.40^*^ [1.04,1.88]	1.30^**^ [1.07,1.58]	1.07 [0.89,1.27]	1.30 [0.94,1.79]	1.02 [0.82,1.27]
Secondary and higher	2.67^***^ [2.27,3.15]	2.34^***^ [1.76,3.12]	1.76^***^ [1.39,2.22]	1.37^**^ [1.11,1.69]	1.67^**^ [1.20,2.33]	1.22 [0.92,1.62]
Religion (Christian)	Ref.	Ref.	Ref.	Ref.	Ref.	Ref.
Muslim	0.79^**^ [0.67,0.93]	0.87 [0.68,1.10]	0.75^**^ [0.59,0.94]	0.99 [0.82,1.20]	1.20 [0.90,1.60]	0.86 [0.66,1.12]
Indigenous beliefs	0.42^***^ [0.35,0.49]	0.42^***^ [0.26,0.67]	0.57^***^ [0.47,0.70]	0.78^*^ [0.64,0.95]	0.74 [0.44,1.24]	0.73^**^ [0.59,0.92]
Ethnicity (Adja-ewe/Mina)	Ref.	Ref.	Ref.	Ref.	Ref.	Ref.
Kabye/Tem	0.69^***^ [0.59,0.82]	0.97 [0.74,1.26]	0.78^*^ [0.62,0.99]	0.93 [0.75,1.15]	1.20 [0.87,1.64]	0.75 [0.56,1.01]
Akposso/Akebou	0.98 [0.70,1.37]	1.64 [0.85,3.17]	1.14 [0.76,1.72]	1.24 [0.86,1.77]	1.64 [0.82,3.29]	1.02 [0.65,1.57]
Ana-ife	0.89 [0.60,1.31]	0.35 [0.13,0.95]	1.52 [0.98,2.37]	1.21 [0.80,1.84]	0.42 [0.15,1.18]	1.41 [0.88,2.25]
Para-gourma/Akan	0.49^***^ [0.41,0.58]	0.73 [0.53,1.00]	0.62^***^ [0.49,0.78]	0.96 [0.74,1.24]	1.00 [0.67,1.48]	0.84 [0.59,1.19]
Other	0.41^***^ [0.26,0.65]	0.92 [0.39,2.18]	0.43^**^ [0.24,0.78]	0.69 [0.41,1.16]	1.45 [0.53,3.96]	0.51^*^ [0.27,0.98]
Non-togolese	1.17 [0.87,1.57]	0.88 [0.62,1.25]	1.41 [0.80,2.47]	0.98 [0.71,1.34]	0.88 [0.60,1.27]	1.41 [0.79,2.51]
Residence (Urban)	Ref.	Ref.	Ref.	Ref.	Ref.	Ref.
Rural	0.38^***^ [0.33,0.44]			1.61^**^ [1.16,2.22]		
Region (Grande agglomération de Lomé)	Ref.	Ref.	Ref.	Ref.	Ref.	Ref.
Maritime	0.38^***^ [0.30,0.49]	0.50^*^ [0.29,0.88]		0.62^**^ [0.45,0.86]	0.64 [0.36,1.16]	
Plateaux	0.40^***^ [0.33,0.49]	0.48^***^ [0.31,0.73]	1.05 [0.80,1.39]	0.65^**^ [0.49,0.87]	0.50^**^ [0.32,0.80]	1.15 [0.83,1.57]
Centrale	0.39^***^ [0.32,0.49]	0.58^**^ [0.40,0.83]	0.96 [0.71,1.29]	0.65^**^ [0.48,0.89]	0.59^*^ [0.38,0.92]	1.27 [0.86,1.86]
Kara	0.29^***^ [0.23,0.36]	0.49^***^ [0.31,0.76]	0.70^*^ [0.51,0.95]	0.56^***^ [0.40,0.77]	0.46^**^ [0.28,0.76]	1.10 [0.75,1.63]
Savanes	0.23^***^ [0.19,0.28]	0.39^***^ [0.25,0.60]	0.57^***^ [0.43,0.76]	0.49^***^ [0.35,0.69]	0.44^**^ [0.26,0.75]	0.89 [0.58,1.36]
Health insurance (No)	Ref.	Ref.	Ref.	Ref.	Ref.	Ref.
Yes	2.96^***^ [2.19,3.98]	2.45^***^ [1.63,3.67]	2.27^***^ [1.41,3.66]	1.93^***^ [1.39,2.67]	2.07^***^ [1.33,3.22]	1.69^*^ [1.02,2.79]
Parity (1–2)	Ref.	Ref.	Ref.	Ref.	Ref.	Ref.
3–4	0.77^***^ [0.66,0.90]	0.70^**^ [0.55,0.88]	0.96 [0.77,1.18]	0.92 [0.78,1.09]	0.77 [0.60,1.00]	1.06 [0.85,1.33]
> 4	0.54^***^ [0.46,0.63]	0.46^***^ [0.34,0.64]	0.82 [0.67,1.00]	0.91 [0.75,1.09]	0.63^*^ [0.44,0.91]	1.06 [0.84,1.32]
Last child wanted (No)	Ref.	Ref.	Ref.			
Yes	1.09 [0.83,1.42]	1.41 [0.96,2.07]	1.05 [0.72,1.54]			
Currently employed (No)	ref	ref	ref			
Yes	0.90 [0.76,1.06]	0.94 [0.73,1.22]	0.99 [0.78,1.26]			
Wealth index (Poorest)	Ref.	Ref.	Ref.	Ref.	Ref.	Ref.
Poorer	1.31^*^ [1.05,1.63]	0.39 [0.05,2.77]	1.33^*^ [1.07,1.66]	1.06 [0.83,1.34]	0.25 [0.03,1.92]	1.07 [0.84,1.37]
Middle	1.41^**^ [1.13,1.74]	0.13^*^ [0.03,0.64]	1.51^***^ [1.22,1.88]	1.03 [0.80,1.32]	0.06^***^ [0.01,0.34]	1.12 [0.86,1.45]
Richer	2.41^***^ [1.96,2.96]	0.82 [0.24,2.83]	2.90^***^ [2.08,4.03]	1.90^***^ [1.35,2.67]	0.34 [0.09,1.27]	1.90^***^ [1.29,2.78]
Richest	5.24^***^ [4.29,6.41]	1.87 [0.54,6.45]	7.88^***^ [2.55,24.32]	3.46^***^ [2.32,5.16]	0.56 [0.15,2.14]	5.69^**^ [1.75,18.49]

Odds ratios with 95% confidence intervals in brackets. Level of significance = ^*^ *p* < 0.05, ^**^ *p* < 0.01, ^***^ *p* < 0.001

However, findings from this study also showed that women who had indigenous beliefs especially in the rural areas had lower odds of timing of first ANC visits (OR = 0.78, 95% CI = 0.64, 0.95) compared to those who were Christians. Regarding ethnicity, the odds of early first ANC visits were significantly lower among women who were from other ethnicities, especially in the rural areas (OR = 0.51, 95% CI = 0.27, 0.98) compared to those from Adja-ewe/Mina ethnicity. As for regional differences, in general, women who were from all the regions especially in the urban areas had lower odds of timely first ANC visits compared to those from Grande Agglomération de Lomé. Concerning the parity, we found that women with higher parity (> 4), especially in the urban areas had lower odds of timing of first ANC visit (OR = 0.63, 95% CI = 0.44, 0.91) compared to those with lower (1–2) parity.

### Factors associated with adequacy of ANC visits in Togo

Table 3 shows the factors associated with adequacy of ANC visits in Togo. In the bivariate analysis, all variables with p-values < 0.05 were selected and added to the multivariable logistic regression model. In this model, we found that women in the age-group 25–34 years and 35–49 years had increased odds of attending adequate number of ANC visits (OR = 1.53, 95% CI = 1.27,1.84) and (OR = 1.61, 95% CI = 1.27,2.05) respectively compared to those aged 15–24 years. Compared to women with no formal education, those who had primary and secondary/higher education especially in both urban and rural areas had increased odds of attending adequate ANC visits (OR = 1.24, 95% CI = 1.06,1.45), and (OR = 1.73, 95% CI = 1.41,2.12) respectively. Being covered by health insurance especially in the urban areas had increased odds of attending adequate number of ANC visits (OR = 1.52, 95% CI = 1.03,2.24) compared to those who were not covered with any health insurance. Similarly, being currently employed was positively associated with higher odds of receiving adequate ANC visits (OR = 1.31, 95% CI = 1.11,1.55) compared to those who were unemployed. Also, the odds of attending adequate ANC visits were significantly higher among women who were from the rich and richest wealth quintile (OR = 1.78, 95% CI = 1.29,2.45), and (OR = 2.19, 95% CI = 1.48,3.24) respectively compared to those from the poorest wealth quintile. Upon stratification by areas, this was true for rural women only.

**Table 3:** Factors associated with adequacy of ANC visits in Togo

	Bivariate analysis (OR, 95%CI)			Multivariable analysis (OR, 95%CI)		
	Overall	Urban	Rural	Overall	Urban	Rural
Adequacy of ANC						
Age groups (15–24)	Ref.	Ref.	Ref.	Ref.	Ref.	Ref.
25–34	1.31^***^ [1.13,1.51]	1.28 [0.95,1.71]	1.24^*^ [1.04,1.48]	1.53^***^ [1.27,1.84]	1.42^*^ [1.02,1.96]	1.55^***^ [1.23,1.94]
35–49	1.06 [0.90,1.24]	1.10 [0.78,1.54]	1.10 [0.91,1.33]	1.61^***^ [1.27,2.05]	1.79^*^ [1.15,2.81]	1.54^**^ [1.15,2.05]
Education (no education)	Ref.	Ref.	Ref.	Ref.	Ref.	Ref.
Primary	1.56^***^ [1.36,1.78]	1.39^*^ [1.03,1.87]	1.40^***^ [1.20,1.64]	1.24^**^ [1.06,1.45]	1.36 [0.98,1.88]	1.24^*^ [1.03,1.48]
Secondary and higher	2.72^***^ [2.32,3.20]	2.37^***^ [1.74,3.23]	1.95^***^ [1.58,2.40]	1.73^***^ [1.41,2.12]	1.92^***^ [1.34,2.77]	1.67^***^ [1.30,2.15]
Religion (Christian)	Ref.	Ref.	Ref.	Ref.	Ref.	Ref.
Muslim	0.86 [0.74,1.01]	0.88 [0.67,1.15]	0.88 [0.73,1.07]	1.03 [0.86,1.23]	1.09 [0.79,1.51]	0.99 [0.79,1.23]
Indigenous beliefs	0.47^***^ [0.41,0.54]	0.53^**^ [0.35,0.81]	0.60^***^ [0.52,0.71]	0.75^***^ [0.64,0.88]	0.88 [0.55,1.42]	0.72^***^ [0.60,0.86]
Ethnicity (Adja-ewe/Mina)	Ref.	Ref.	Ref.	Ref.	Ref.	Ref.
Kabye/Tem	0.74^***^ [0.64,0.87]	1.02 [0.76,1.38]	0.85 [0.69,1.04]	0.88 [0.72,1.08]	1.14 [0.80,1.64]	0.78 [0.60,1.01]
Akposso/Akebou	0.86 [0.63,1.20]	1.27 [0.57,2.82]	1.03 [0.71,1.50]	1.15 [0.81,1.64]	1.30 [0.57,2.97]	1.04 [0.70,1.56]
Ana-ife	0.67^*^ [0.46,0.96]	0.40^*^ [0.18,0.91]	0.98 [0.64,1.49]	0.90 [0.60,1.33]	0.52 [0.22,1.26]	0.99 [0.63,1.54]
Para-gourma/Akan	0.59^***^ [0.50,0.69]	1.14 [0.79,1.64]	0.71^***^ [0.58,0.86]	1.15 [0.90,1.47]	1.30 [0.83,2.05]	1.09 [0.81,1.48]
Other	0.61^**^ [0.43,0.88]	0.73 [0.29,1.81]	0.79 [0.53,1.18]	1.02 [0.67,1.54]	0.97 [0.36,2.61]	0.96 [0.60,1.54]
Non-togolese	1.08 [0.80,1.47]	0.86 [0.59,1.26]	1.01 [0.60,1.72]	0.91 [0.66,1.26]	0.91 [0.61,1.37]	0.97 [0.56,1.67]
Residence (Urban)	Ref.	Ref.	Ref.	Ref.	Ref.	Ref.
Rural	0.39^***^ [0.34,0.45]			1.04 [0.76,1.42]		
Region (Grande agglomération de Lomé)	Ref.	Ref.	Ref.	Ref.	Ref.	Ref.
Maritime	0.50^***^ [0.39,0.63]	0.86 [0.47,1.54]		0.98 [0.70,1.35]	0.91 [0.49,1.69]	
Plateaux	0.38^***^ [0.31,0.46]	0.49^***^ [0.33,0.74]	0.78 [0.61,1.00]	0.74^*^ [0.55,0.99]	0.50^**^ [0.32,0.78]	0.79 [0.60,1.04]
Centrale	0.50^***^ [0.41,0.63]	0.89 [0.59,1.33]	0.96 [0.74,1.24]	0.96 [0.70,1.32]	0.91 [0.56,1.48]	1.01 [0.72,1.42]
Kara	0.40^***^ [0.32,0.50]	0.73 [0.46,1.15]	0.79 [0.61,1.02]	0.89 [0.65,1.23]	0.74 [0.44,1.25]	0.94 [0.67,1.31]
Savanes	0.28^***^ [0.23,0.34]	0.80 [0.51,1.25]	0.55^***^ [0.44,0.70]	0.63^**^ [0.45,0.88]	0.95 [0.54,1.68]	0.60^*^ [0.42,0.86]
Health insurance (No)	Ref.	Ref.	Ref.	Ref.	Ref.	Ref.
Yes	2.70^***^ [1.87,3.90]	2.87^***^ [1.56,5.30]	1.78^*^ [1.09,2.89]	1.52^*^ [1.03,2.24]	2.10^*^ [1.10,4.00]	1.19 [0.72,1.98]
Parity (1–2)	Ref.	Ref.	Ref.	Ref.	Ref.	Ref.
3–4	0.80^**^ [0.69,0.93]	0.94 [0.72,1.24]	0.82^*^ [0.69,0.98]	0.78^**^ [0.65,0.94]	0.94 [0.68,1.29]	0.72^**^ [0.58,0.90]
> 4	0.60^***^ [0.52,0.69]	0.43^***^ [0.31,0.59]	0.83^*^ [0.70,0.98]	0.76^*^ [0.61,0.94]	0.49^***^ [0.32,0.76]	0.82 [0.64,1.07]
Last child wanted (No)	Ref.	Ref.	Ref.	Ref.	Ref.	Ref.
Yes	1.16 [0.92,1.47]	1.75^**^ [1.19,2.57]	1.08 [0.80,1.47]	1.26 [0.98,1.64]	1.44 [0.93,2.23]	1.11 [0.80,1.53]
Currently employed (No)	Ref.	Ref.	Ref.	Ref.	Ref.	Ref.
Yes	1.13 [0.96,1.32]	1.20 [0.90,1.59]	1.23^*^ [1.01,1.50]	1.31^**^ [1.11,1.55]	1.27 [0.93,1.73]	1.35^**^ [1.09,1.66]
Wealth index (Poorest)	Ref.	Ref.	Ref.	Ref.	Ref.	Ref.
Poorer	1.24^*^ [1.05,1.48]	2.10 [0.38,11.59]	1.24^*^ [1.04,1.47]	1.07 [0.89,1.30]	1.54 [0.26,8.95]	1.03 [0.85,1.25]
Middle	1.33^***^ [1.12,1.58]	2.01 [0.53,7.62]	1.33^***^ [1.12,1.59]	1.02 [0.83,1.25]	1.89 [0.47,7.52]	0.99 [0.80,1.22]
Richer	2.70^***^ [2.24,3.26]	4.30^*^ [1.24,14.87]	2.96^***^ [2.10,4.16]	1.78^***^ [1.29,2.45]	3.34 [0.90,12.36]	1.77^**^ [1.21,2.59]
Richest	4.09^***^ [3.34,5.02]	6.82^**^ [1.97,23.60]	1.70 [0.55,5.23]	2.19^***^ [1.48,3.24]	4.04^*^ [1.07,15.24]	0.83 [0.25,2.68]

Odds ratios with 95% confidence intervals in brackets. Level of significance = ^*^ *p* < 0.05, ^**^ *p* < 0.01, ^***^ *p* < 0.001

However, our results also showed that the probability of attending adequate ANC visits were significantly lower among women who had indigenous beliefs especially in the rural areas (OR = 0.75, 95% CI = 0.64,0.88) compared to those who were Christians. Also, women who were from Plateaux and Savanes regions had lower odds of attending adequate ANC visits (OR = 0.74, 95% CI = 0.55,0.99), and (OR = 0.63, 95% CI = 0.45,0.88) respectively compared to those from Grande Agglomération de Lomé. Lastly, having 3–4, and > 4) parity had lower odds of attending adequate ANC visits (OR = 0.78, 95% CI = 0.65,0.94), and (OR = 0.76, 95% CI = 0.61,0.94) respectively compared to those with lower parity.

### Factors associated with health facility delivery in Togo

Table 4 shows the factors associated with health facility delivery in Togo. In this Table, results of a bivariate analysis are presented and all factors with p-values < 0.05 were selected and added to the multivariable logistic regression model. Women aged 25–34 years and 35–49 years especially in the rural areas had higher odds of delivering in a health facility (OR = 1.48, 95% CI = 1.15,1.91), and (OR = 1.59, 95% CI = 1.16,2.18) respectively compared to those aged 15–24 years. Also, women with primary and secondary / higher level of education in both urban and rural areas had higher odds of delivering in a health facility (OR = 1.62, 95% CI = 1.34,1.96), and (OR = 3.47, 95% CI = 2.55,4.73) respectively compared to those with no formal education. Concerning health insurance, we found that women who were covered by health insurance especially in the rural areas had 2.36 times higher odds of delivering in a health facility (OR = 2.36, 95% CI = 1.12,4.94) compared to those who were not covered by health insurance. For occupation status, we found that currently employed women in the urban areas only had higher odds of delivering in a health facility (OR = 2.18, 95% CI = 1.16,4.09) compared to those who were unemployed. We found also that belonging to the rich and richest wealth quintile was significantly associated with higher odds of delivering in a health facility (OR 6.75, 95% CI = 4.05,11.30), and (OR = 8.53, 95% CI = 4.06,17.92) respectively compared to those from the poorest wealth quintile.

**Table 4:** Factors associated with healthcare facility for delivery in Togo

	Bivariate analysis (OR, 95%CI)			Multivariable analysis (OR, 95%CI)		
	Overall	Urban	Rural	Overall	Urban	Rural
Healthcare facility						
Age groups (15–24)	Ref.	Ref.	Ref.	Ref.	Ref.	Ref.
25–34	1.01 [0.85,1.21]	0.91 [0.46,1.79]	0.91 [0.75,1.11]	1.48^**^ [1.15,1.91]	1.19 [0.53,2.64]	1.48^**^ [1.13,1.94]
35–49	0.67^***^ [0.55,0.80]	0.75 [0.35,1.60]	0.68^***^ [0.56,0.84]	1.59^**^ [1.16,2.18]	1.65 [0.58,4.67]	1.56^**^ [1.12,2.17]
Education (no education)	Ref.	Ref.	Ref.	Ref.	Ref.	Ref.
Primary	2.66^***^ [2.28,3.10]	2.05^**^ [1.19,3.53]	2.24^***^ [1.89,2.64]	1.62^***^ [1.34,1.96]	1.42 [0.75,2.67]	1.61^***^ [1.32,1.97]
Secondary and higher	9.59^***^ [7.38,12.46]	10.24^***^ [4.22,24.88]	5.62^***^ [4.23,7.46]	3.47^***^ [2.55,4.73]	5.45^***^ [2.02,14.77]	3.21^***^ [2.32,4.46]
Religion (Christian)	Ref.	Ref.	Ref.	Ref.	Ref.	Ref.
Muslim	0.90 [0.74,1.10]	0.30^***^ [0.17,0.53]	1.11 [0.89,1.39]	1.36^*^ [1.07,1.74]	0.58 [0.29,1.15]	1.61^***^ [1.24,2.10]
Indigenous beliefs	0.22^***^ [0.19,0.26]	0.20^***^ [0.09,0.42]	0.36^***^ [0.30,0.42]	0.59^***^ [0.49,0.71]	0.53 [0.22,1.30]	0.61^***^ [0.50,0.75]
Ethnicity (Adja-ewe/Mina)	Ref.	Ref.	Ref.	Ref.	Ref.	Ref.
Kabye/Tem	0.46^***^ [0.37,0.56]	0.34^**^ [0.16,0.69]	0.68^***^ [0.54,0.86]	0.73^*^ [0.55,0.97]	0.57 [0.24,1.31]	0.78 [0.57,1.06]
Akposso/Akebou	0.40^***^ [0.27,0.58]	0.36 [0.08,1.64]	0.63^*^ [0.42,0.95]	0.58^*^ [0.38,0.90]	0.36 [0.07,1.74]	0.62^*^ [0.39,0.97]
Ana-ife	0.48^***^ [0.31,0.75]	0.46 [0.06,3.64]	0.79 [0.49,1.27]	0.87 [0.52,1.45]	0.70 [0.08,5.95]	0.89 [0.53,1.52]
Para-gourma/Akan	0.21^***^ [0.17,0.25]	0.26^***^ [0.12,0.55]	0.34^***^ [0.27,0.42]	0.59^***^ [0.43,0.82]	0.43 [0.16,1.14]	0.62^**^ [0.44,0.88]
Other	0.26^***^ [0.18,0.39]	0.13^**^ [0.03,0.48]	0.46^***^ [0.30,0.70]	0.60^*^ [0.37,0.98]	0.28 [0.06,1.36]	0.61 [0.36,1.03]
Non-togolese	1.25 [0.78,2.00]	0.34^*^ [0.14,0.81]	1.06 [0.56,2.01]	0.74 [0.43,1.26]	0.48 [0.19,1.23]	1.06 [0.53,2.13]
Residence (Urban)	Ref.	Ref.	Ref.	Ref.	Ref.	Ref.
Rural	0.09^***^ [0.07,0.11]			0.93 [0.57,1.53]		
Region (Grande agglomération de Lomé)	Ref.	Ref.	Ref.	Ref.	Ref.	Ref.
Maritime	0.19^***^ [0.11,0.30]			0.98 [0.51,1.86]		
Plateaux	0.07^***^ [0.05,0.11]	0.31^**^ [0.14,0.67]	0.39^***^ [0.28,0.53]	0.39^**^ [0.21,0.72]	0.27^**^ [0.11,0.67]	0.42^***^ [0.29,0.60]
Centrale	0.11^***^ [0.07,1.18]	0.52 [0.22,1.23]	0.58^**^ [0.41,0.81]	0.63 [0.34,1.18]	1.11 [0.40,3.08]	0.62^*^ [0.41,0.95]
Kara	0.05^***^ [0.03,0.08]	0.25^***^ [0.12,0.54]	0.26^***^ [0.19,0.36]	0.50^*^ [0.27,0.93]	0.48 [0.19,1.24]	0.51^**^ [0.34,0.78]
Savanes	0.04^***^ [0.02,0.05]	0.24^***^ [0.12,0.51]	0.20^***^ [0.15,0.26]	0.62 [0.33,1.18]	0.88 [0.31,2.50]	0.64^*^ [0.41,0.99]
Health insurance (No)	Ref.	Ref.	Ref.	Ref.	Ref.	Ref.
Yes	6.45^***^ [3.29,12.65]	4.98 [0.68,36.29]	4.35^***^ [2.08,9.10]	2.36^*^ [1.12,4.94]	2.62 [0.33,20.80]	2.29^*^ [1.03,5.11]
Parity (1–2)	Ref.	Ref.	Ref.	Ref.	Ref.	Ref.
3–4	0.62^***^ [0.52,0.74]	0.45^*^ [0.24,0.83]	0.70^***^ [0.58,0.86]	0.68^**^ [0.53,0.88]	0.55 [0.26,1.16]	0.72^*^ [0.55,0.94]
> 4	0.31^***^ [0.26,0.37]	0.26^***^ [0.13,0.50]	0.46^***^ [0.38,0.55]	0.62^***^ [0.47,0.83]	0.44 [0.17,1.11]	0.66^**^ [0.49,0.88]
Last child wanted (No)	Ref.	Ref.	Ref.			
Yes	1.04 [0.79,1.36]	1.36 [0.61,3.06]	1.28 [0.94,1.74]			
Currently employed (No)	Ref.	Ref.	Ref.	Ref.	Ref.	Ref.
Yes	0.62^***^ [0.51,0.76]	1.94^*^ [1.12,3.35]	0.59^***^ [0.47,0.74]	0.90 [0.71,1.14]	2.18^*^ [1.16,4.09]	0.78 [0.61,0.99]
Wealth index (Poorest)	Ref.	Ref.	Ref.	Ref.	Ref.	Ref.
Poorer	2.12^***^ [1.77,2.54]	1.52 [0.25,9.29]	2.13^***^ [1.78,2.55]	1.60^***^ [1.31,1.97]	1.01 [0.14,7.02]	1.62^***^ [1.32,1.99]
Middle	4.68^***^ [3.81,5.75]	3.11 [0.75,12.86]	4.66^***^ [3.77,5.75]	2.67^***^ [2.09,3.40]	2.29 [0.47,11.22]	2.70^***^ [2.11,3.46]
Richer	19.80^***^ [14.05,27.92]	11.09^***^ [3.08,39.96]	22.41^***^ [10.95,45.89]	6.75^***^ [4.04,11.30]	5.96^*^ [1.31,27.21]	8.30^***^ [3.95,17.44]
Richest	44.67^***^ [27.29,73.12]	27.07^***^ [7.20,101.80]	11.89^***^ [1.54,91.70]	8.53^***^ [4.06,17.92]	8.39^**^ [1.65,42.56]	2.04 [0.25,16.15]

Odds ratios with 95% confidence intervals in brackets. Level of significance = ^*^ *p* < 0.05, ^**^ *p* < 0.01, ^***^ *p* < 0.001

However, findings from this study also showed that the odds of delivering at a health facility were significantly lower among women with indigenous beliefs (OR = 0.59, 95% CI = 0.49,071) and higher among those who were Muslin in their faith (OR = 1.36, 95% CI = 1.07,1.74) compared to those who were Christians. Stratified by areas, this association was significant in the rural areas only. Lower odds of delivering in a health facility were also found among women who were from Kabye/Tem (OR = 0.73, 95% CI = 0.55,0.97), Akposso/Akebou (OR = 0.58, 95% CI = 0.38,0.90), Para-gourma/Akan (OR = 0.59, 95% CI = 0.43,0.82) and other (OR = 0.60, 95% CI = 0.37,0.98) ethnicities compared to those from Adja-ewe/Mina ethnicity. Compared to residents of Grande Agglomeration de Lomé, those in other regions (except for Maritime and Centrale region) had lower odds of delivering at a health facility. This regional difference was true only for rural residents, except for Maritime region. Also having higher parity (> 4) was significantly associated with lower odds of delivering in a health facility (OR = 0.62, 95% CI = 0.47,0.83) compared to those of lower (1–2) parity. This association was similar for rural women only (OR = 0.66, 95% CI = 0.49,088).

## Discussion

This cross-sectional study identified the proportion and factors associated with utilization of maternal healthcare services (timing of first ANC visits, adequate ANC visits, and healthcare facility delivery), using the 2013 TDHS-3 dataset.

The overall proportion of maternal healthcare utilization was 27.53% for timing of first ANC visits, 59.99% for adequate ANC visits, and 75.66% for delivery at a healthcare facility. These findings agrees with results of previous cross-sectional studies conducted in Ethiopia [33], and Cameroon [34] in which a higher proportion of timing of initiation of ANC visits was reported. Regarding adequate number of ANC visits, our finding is similar to findings of the systematic review conducted in Nepal [35] and dissimilar to a cross-sectional study done in Bangladesh [29]. In terms of health facility use, consistent findings are also reported from cross-sectional studies conducted in Ghana [36], and Senegal [37].These differences could be explained by socio-economic inequalities, socio-cultural barriers, limited and poorly equipped health facilities and poor quality of health services offered to women. There is a need to equip healthcare facilities, improve health service quality, educate women on the utilization of these facilities for delivery services and a need for women empowerment.

In our multivariable model, we found that age, education, religion, ethnicity, socioeconomic status, urban vs rural residence, region of residence, parity, and health insurance are variables that can help explain differential access to maternal healthcare services in Togo. For instance, women with higher age compared to those with lower age had higher odds of using the maternal healthcare services, except for timing of initiation of ANC visits. These findings are largely similar to those in other low and middle-income countries where advanced maternal age showed a positive association with the attendance of ANC4 + visits [38] and delivering in a health facility [39]. This is perhaps because, pregnant women with higher ages are at higher risk of experiencing complications during delivery, hence, they are more knowledgeable about the maternal healthcare services. In contrast, pregnant women with younger ages might be more immature and might have limited information about pregnancy and the utilization of prenatal and postnatal care services.

Also, this study’s findings adds to those of previous studies showing that with increase education there is a greater likelihood of a timely first ANC visit [33, 40], having adequate ANC visits [41, 42], and delivering at a health facility [43, 44], compared to those with no formal education. The possible reason for this could be that educated women are more informed and can make better decisions in favor of attending ANC visits and using health facility delivery services. Evidence further shows that one year of schooling for a mother reduces the risk of death in children under 5 by 3% [45]. As such, women education needs to be promoted in order to achieve sustainable maternal health.

In the current study, significantly lower odds of early first ANC visits was found among women from other ethnicities compared to those from Adja-ewe/Mina ethnicity. Beside this, we also found lower odds of delivering in a health facility among women from Kabye/Tem, Akposso/Akebou, Para-gourma and other ethnicities compared to those from Adja-ewe and Mina ethnicity. This finding was supported by other previous studies in which maternal ethnicity was found to be a predictive factor of underuse of maternal healthcare services [46, 47]. These ethnic differences could be due to women’s lack of confidence and understanding of the maternal healthcare services, and other cultural factors that can impact women’s healthcare seeking behavior.

Our study also found that women who were covered by health insurance especially in the rural areas had increased odds of attending the first ANC visits within the first trimester, receiving adequate ANC visits, and delivering at health facility compared to those who were not covered by health insurance. These findings agree with the findings of a study conducted in Jordan in which health insurance coverage was significantly associated with higher odds of utilizing maternal healthcare services [48]. This could be because women without health insurance face financial barriers to accessing healthcare.

Furthermore, socioeconomic factors were also found to be significantly associated with the odds of using maternal healthcare services. For instance, women in the rich and richest wealth quintile, especially in the rural areas had higher odds of timely first ANC visits, attending adequate ANC visits and delivering at health facility compared to those from the poorest wealth quintile. This aligns with previous studies done in Ethiopia [49], Gabon [9], and Ghana [50]. One explanation of this could be that women of higher socioeconomic status have the financial resources to seek health compared to those of lower-income families [51]. We perceive that there is a need to promote women’s economic empowerment to improve utilization of maternal healthcare services.

For occupational status, women who were currently employed compared to those who were unemployed showed a positive effect on having adequate ANC visits, and health facility delivery. These were supported by findings from previous studies that reported that employed women were more likely than unemployed women to attend ANC4 + visits [52], and deliver at health facility [53]. This could be because currently employed women have the financial means to afford health care services for their well-being and that of their future babies.

In this study, area of residence and regional differences played an important role in the odds of utilizing maternal healthcare services. As such, women in the rural areas had higher odds of timely first ANC visits compared to those in the urban areas. For adequate ANC visits and health facility, area of residence did not show any significant results. These findings are well in line with the literature indicating that women especially those in the rural areas were less likely to have ANC4 + visits [54–56], and deliver at a health facility [57, 58]. We perceive that this could be attributed to socio-economic disparities and socio-cultural factors—rural woman are financially dependent on their husband / partner and have lower income, they are less educated and are less informed on the importance of accessing maternal healthcare services than those from urban areas.

Similarly, we also observed the lowest odds of using maternal healthcare services among women from all other regions compared to those from Grande agglomeration de Lomé region. Stratified by areas, this association is similar in the urban areas and dissimilar in the rural areas. This might be due to the regional disparities in access to utilization of healthcare services.

Interestingly, our model also showed that women with indigenous beliefs especially in rural areas had lower odds of timing of first ANC visits, having adequate ANC visit and delivering in a health facility compared to those who were Christians. In contrast, women who were Muslim had higher odds of delivering at a health facility compared to those who were Christians. Although there is no clear explanation for this, a study has suggested that a number of Muslim women are betrothed to influential Muslim men which compels and facilitates the women to seek and access ANC services [57].

In addition to this, our study also showed a negative association between higher parity and all outcome variables. Evidence from previous studies showed that higher parity was significantly associated with lower odds of early booking of ANC visits [59], attending adequate ANC visits [60, 61], and delivering at a health facility [62]. Also, studies by Mugambe et al. and Gitonga and Muiruri done in Uganda and Kenya respectively identified having higher parity as a predictive factor of women’s under-use of health facility delivery [63, 64]. The possible justification may be that woman with prior experience with delivery were less motivated to use ANC visits or deliver at health facility which have been reported previously in other studies [65, 66].

## Strengths and limitations

The main strength of this study is the use of nationally representative datasets – DHS-3; therefore, the results can be generalized especially for women aged 15–49 years in the whole country, although the sample size may not be large enough for the analysis of some subcategories. To the best of our knowledge, this study is the first to analyze the social determinants affecting the use of maternal healthcare services in Togo using DHS-3 datasets. However, this study has a few limitations to report. Firstly, a cross-sectional design was used, and this makes it hard to establish causal relationships from the associations between explanatory and outcome variables. Secondly, this study used secondary data and all the information given by the respondents about the outcome variables (such as timing and number of ANC visits, and health facility) and explanatory variables (such as last child wanted) were based on self-reported information, hence the chances of recall and social desirability biases abound.

## Conclusion

Overall, our findings indicate that advanced maternal age, maternal education, health insurance, having an occupation, and being part of the rich and richest wealth index are significantly associated with increased odds of using maternal healthcare services. Apart from women who lived in the rural areas, region of residence, ethnicity, having indigenous beliefs, as well as having higher parity was negatively associated with the likelihood of utilizing all the maternal healthcare services. These findings warrant that future research should be done to establish the causality links between social determinants of health and utilization of maternal healthcare services. Our results suggest that there is a need to improve access to, and the utilization of maternal healthcare services in Togo by addressing socio-economic and socio-cultural barriers.

## Data Availability

The dataset is freely accessible for download from the Demographic and Health Surveys program (https://dhsprogram.com/data/dataset/Togo_Standard-DHS_2013.cfm?flag=0).

## Abbreviations

ANC: Antenatal care
CI: Confidence interval
ICF: International Coaching Federation
INSEED: National Institute of Statistics Economical and Demographic
LMICs: Low- or middle-income countries
OR: Odds ratio
SDGs: Sustainable Development Goals
TDHS: Togo Demographic and Health Survey
UN: United Nations
USAID: United States Agency for International Development
VIF: Variance Inflation Factor
WHO: World Health Organization
